# Psychoanalytische Erkundungen der Gesellschaft: Möglichkeiten und Grenzen einer psychoanalytischen Sozialforschung

**DOI:** 10.1007/s00451-022-00480-x

**Published:** 2022-10-28

**Authors:** Markus Brunner, Florian Knasmüller, Julia König

**Affiliations:** 1 Sigmund Freud PrivatUniversität, Wien, Österreich; 2 Institut für Erziehungswissenschaft, Johannes Gutenberg Universität, Mainz, Deutschland

## Abstract

Die psychoanalytische Erkundung gesellschaftlicher Phänomene beginnt bereits mit Freuds kulturtheoretischen Schriften. In diesem Artikel zeichnen wir nach, wie an Freud anschließende kritische Denker:innen die Psychoanalyse für Gesellschaftsanalysen produktiv zu machen suchten, und entwickeln davon ausgehend Konturen einer psychoanalytisch orientierten Sozialforschung, die nicht nur psychoanalytische Erkenntnisse „anwendet“, sondern der gesellschaftlichen Hervorbringung innerpsychischer Konflikte nachspürt. Wir gehen dann auf Alfred Lorenzers Methodologie und Methode der Tiefenhermeneutischen Kulturanalyse ein und geben aus dieser theoretischen Perspektive einen Einblick in aktuelle Forschungen zu den Coronaprotesten.

## Abstract (en)

The psychoanalytic exploration of social phenomena goes back as far as Freudʼs writings on cultural theory. In this article we aim at delineating how critical scholars following Freud sought to make psychoanalysis fruitful for social analyses and, based on this, develop contours of psychoanalytically oriented social research. This school of thought does not simply “apply” psychoanalytic knowledge but traces the social production of psychological conflicts. Moreover, we introduce Alfred Lorenzerʼs methodology and method of in-depth hermeneutic cultural analysis and from this theoretical perspective provide insights into recent research on the coronavirus disease 2019 (COVID-19) protests.

## Einleitung

Die Psychoanalyse wird fast seit ihrem Bestehen von der Versuchung begleitet, ihre in der therapeutischen, klinischen Praxis gewonnenen Begriffe und Konzepte auf gesellschaftliche Konstellationen zu übertragen. Bereits Sigmund Freud war bestrebt, nicht nur die Analysand:innen auf seiner Couch zu analysieren und die Gesetze des psychischen Apparats zu beschreiben, sondern er analysierte in seinen kulturtheoretischen Schriften auch gesellschaftliche Phänomene, wie Religion, Krieg, Massen und den Antisemitismus, sowie auch sehr grundlegend das Verhältnis von Individuum und Gesellschaft, die bei Freud immer als *Kultur* erscheint. Psychoanalytische Erkundungen der Gesellschaft begegnen uns auch heute immer wieder, oft etwa als Zeitdiagnosen mit dem Anspruch, *die Gesellschaft *als solche unter die Lupe zu nehmen beziehungsweise auf die Couch zu legen: Von *narzisstischen Gesellschaften* oder der *Wiederkehr des Verdrängten *in gesellschaftlichen Diskursen oder Konflikten ist die Rede, immer wieder werden auch Ferndiagnosen der Persönlichkeiten politischer Akteur:innen vorgelegt. Solche psychoanalytischen Annäherungen an scheinbar unbegreifliche gesellschaftliche Entwicklungen – und das mit diesen einhergehende Unbehagen – können durchaus erhellend sein, indem sie Licht werfen, auf die Funktionalität des vermeintlich Irrationalen und auf unbewusste Dynamiken und Affektlagen, die dem soziologischen Blick zwangsläufig entgehen müssen. Vorsicht geboten ist dabei jedoch, weil derartige Erklärungsversuche auch dazu tendieren, die Eigenlogik gesellschaftlicher Vorgänge zu unterschlagen, sofern das psychoanalytische Instrumentarium bruchlos auf diese angewendet wird.

In den folgenden Ausführungen wollen wir herausarbeiten, dass eine psychoanalytisch orientierte Sozialforschung einen wichtigen Beitrag zu Gesellschaftsanalysen leisten kann, gerade wenn sie sich die Fallstricke bewusst macht, die mit einem solchen Vorhaben einhergehen, und insofern sie weder das Psychische noch die Gesellschaft als ahistorische Größen betrachtet. Eine psychoanalytische Sozialforschung, so werden wir zeigen, muss ihren Gegenstand vielmehr stets als historisch gewordenen und gesellschaftlich (mit-)produzierten begreifen und sie muss gerade die jeweils besonderen Brechungen des Gesellschaftlichen im Subjekt analysieren, will sie ihrem kritischen Anspruch gerecht werden.

Zunächst werden wir Grundzüge der psychoanalytischen Sozialforschung skizzieren und darlegen, auf welchen Grundsätzen und Annahmen sie beruht und wie sie Gesellschaftstheorie und Psychoanalyse zusammendenkt. Es folgt eine Darstellung der von Alfred Lorenzer entworfenen sozialwissenschaftlichen Methodologie und Methode der Tiefenhermeneutischen Kulturanalyse. Danach werden wir anhand der Forschung zu den Protesten gegen Coronamaßnahmen im deutschsprachigen Raum an einem konkreten Anwendungsbeispiel veranschaulichen, worauf eine Forschung, die die methodologischen Differenzen von therapeutischer Psychoanalyse im Behandlungszimmer und Sozialforschung reflektiert, ihren Blick lenkt, und welchen Fragestellungen sie nachgeht. Dazu zeigen wir an einer Fallanalyse auf, wie latente Affekt- und Konfliktlagen aus empirischem Material herausgearbeitet werden, und wie diese latenten Sinnebenen wiederum als Resultate gesellschaftlicher Kräfteverhältnisse gedeutet werden können.

## Psychoanalytisch orientierte Sozialforschung

Freud spürte in seinem Werk nicht nur den unbewussten Prozessen in gesellschaftlichen Phänomenen nach, sondern es finden sich darin auch erste Auseinandersetzungen mit der Frage der gesellschaftlichen Bedingtheit des Psychischen. In seinem Aufsatz „Die ‚kulturelle‘ Sexualmoral und die moderne Nervosität“ aus dem Jahr 1908 macht Freud bereits auf die gesellschaftlichen Bedingungen von psychischen Erkrankungen aufmerksam. Auch in seinen Krankengeschichten finden sich immer wieder Hinweise darauf, wie die Analysand:innen mit gesellschaftlichen Normen, Anforderungen und Verunmöglichungen ringen, wie also die Kultur die „Triebschicksale“ maßgeblich mitbestimmt. Freud begründete damit im Kern das, was wir heute eine psychoanalytisch orientierte Sozialforschung oder psychoanalytische Sozialpsychologie nennen und was im weiteren Verlauf unterschiedlich weiterentwickelt wurde (zur Geschichte dieser Tradition im deutschsprachigen Raum: Brunner et al. 2012). Freud entwickelte einen ungemein produktiven Blick auf gesellschaftliche Phänomene „vom Seelenende her“ (Freud 1986, S. 294). Hierzu nahm er einerseits die Kosten der Vergesellschaftung in den Blick; er zeigte, was im Sozialisationsprozess verdrängt und verworfen werden musste. Andererseits forschte er nach, wie sich die dabei unbewusst gemachten Regungen gesellschaftlich und in Form von kollektiven Symptombildungen auswirkten. So konnte er den unbewussten Dynamiken des gesellschaftlichen Lebens auf die Spur kommen, die dem sozialwissenschaftlichen Blick entgingen. Dass diese „Psychoanalyse jenseits der Couch“ allerdings auch ihre Fallstricke mit sich brachte, verdeutlichen nicht zuletzt Freuds Schriften selbst: In *Neue Folge der Vorlesungen zur Einführung in die Psychoanalyse* bestimmte er die Soziologie als „nichts anderes … als *angewandte* Psychologie“ (Freud 1933, S. 194) und zeigte damit, dass er wenig Sinn für die Eigenlogiken gesellschaftlicher Dynamiken und Institutionen sowie für die Bedeutung moderner, kapitalistischer Herrschaftsverhältnisse und ihrer Verwerfungen hatte. Mit diesen Ausblendungen wurden soziale Phänomene enthistorisiert und immer wieder als Ausdruck einer generellen Beschaffenheit der menschlichen Psyche gelesen.

Es waren zuerst die am Frankfurter Institut für Sozialforschung angesiedelten Vertreter der frühen Kritischen Theorie, die Ende der 1920er-Jahren bestrebt waren, die Psychoanalyse systematisch für eine kritische Sozialforschung und Gesellschaftstheorie nutzbar zu machen. Der Sozialphilosoph Max Horkheimer formulierte ein umfassendes Programm einer gesellschaftstheoretisch reflektierten empirischen Sozialforschung, in der der Psychoanalyse eine besondere Stellung zukam: Sie sollte angesichts des verheerenden Ersten Weltkriegs, den ausbleibenden oder niedergeschlagenen sozialistischen Revolutionen und der immer stärker werdenden nationalsozialistischen Bewegung erklären helfen, weshalb sich die Arbeiter:innenschaft, anstatt die bestehenden Herrschaftsverhältnisse als Klassenverhältnisse zu bekämpfen und zu überwinden, eher gesellschaftlichen Autoritäten unterwarf und autoritären Bewegungen anschloss (Horkheimer 1932). Die psychoanalytisch orientierte Sozialpsychologie sollte, so Adorno (1955, S. 42) „subjektiven Bedingungen der objektiven Irrationalität“ nachforschen: Sie fragte danach, wie sich Herrschaftsverhältnisse in den Individuen sedimentierten und in Autoritarismus, Ressentiments und Gewalt umsetzten. Dafür war es notwendig, dem komplexen Zusammenspiel von gesellschaftlichen Prozessen, Logiken und Psychodynamiken auf die Spur zu kommen. Das hieß zuallererst, jedes soziale Phänomen, auch wenn es noch so überzeitlich erscheint, als historisch und gesellschaftlich spezifischen Gegenstand zu verstehen und dessen Entstehung und Wandel als Ausdruck einer gesamtgesellschaftlichen Bewegung zu erfassen. Das betraf auch die unbewussten Dimensionen, auf deren Spuren sich die psychoanalytische Sozialforschung im Kontext der sogenannten Frankfurter Schule begab. Die psychoanalytische Sozialpsychologie blickte einerseits auf die „gesellschaftliche Produktion von Unbewusstheit“ (Erdheim 1984) und andererseits auf die gesellschaftlichen Ausdrucksformen der so entstandenen Konfliktlagen. Dieser doppelte Blick hieß immer auch, dezidiert historisch und gesellschaftstheoretisch zu betrachten, wie in verschiedenen Familienstrukturen, die ein Produkt der sozialen Bedingungen sind, aber auch in Institutionen und durch gesellschaftliche Strukturen Subjekte formiert, Wünsche beziehungsweise die Freud’schen Triebe hervorgebracht, geformt, verworfen oder desymbolisiert werden.

Die so hergestellten Konflikte werden nicht nur individuell abgewehrt oder bearbeitet, sondern es gibt auch gesellschaftlich nahegelegte und zur Verfügung gestellte Abwehrformationen, die die individuelle Abwehr stützen. Die Distinktion ermöglichende Identifikation mit Kollektiven (Nationen, Fußballvereinen …), Institutionen (Kirchen, Unternehmen, Universitäten …) und gesellschaftlichen Führer:innen, der Besitz von Gütern oder der ausgestellte Oberschichts- oder Intellektuellen-Habitus oder die Teilhabe an Angeboten der Konsum- und Unterhaltungsindustrie bieten verschiedenartige narzisstische Stützen, Möglichkeiten der imaginären Verschmelzung, der aktiven wie passiven Unterwerfung oder der Aggressionsabfuhr. Kollektive Abgrenzungs- und Feindbildungsprozesse und Bilder der „Anderen“ ermöglichen die projektive Verortung verdrängter Regungen und erlauben die Artikulation sonst verpönter Aggressionen. Diese gesellschaftlichen Angebote können zur individuellen Entlastung genutzt werden – Freud (1921, S. 159) hatte für diesen Entlastungsprozess in seiner Arbeit „Massenpsychologie und Ich-Analyse“ den Begriff der „Schiefheilung“ geprägt: Drohende individuelle Erkrankungen werden dadurch verhindert, dass die innerpsychischen Konflikte ausgelagert werden (Brunner 2015).

Vor dem Hintergrund dieser Ausführungen wird deutlich, dass die reflektierte psychoanalytische Erkundung des gesellschaftlichen Feldes immer wieder von Neuem der Vermittlung von psychoanalytischen mit sozialwissenschaftlichen Erkenntnissen und Reflexionen bedarf: Die radikale Innenperspektive auf die individuellen Wünsche, Bedeutungen, Konfliktlagen und Konfliktbearbeitungsversuche wird einem soziologischen Blick auf gesellschaftliche und institutionelle Kontexte, Funktionen und Widersprüche gegenübergestellt. Beide Perspektiven lassen sich nicht ineinander auflösen, widersprechen sich zuweilen – sie sind als Spannungsverhältnis zu begreifen, das sich nicht harmonisieren lässt, innerhalb dessen sich aber die beiden Perspektiven stets kritisch korrigieren: Weder dürfen Phänomene psychologisiert noch darf soziologistisch die Eigenlogik unbewusster Prozesse außer Acht gelassen werden (Adorno 1955; Lorenzer 1977). In dieser Spannung entfaltet sich aber das große Potenzial der psychoanalytischen Sozialpsychologie als Teil einer kritischen Sozialforschung.

## Alfred Lorenzer und die Tiefenhermeneutische Kulturanalyse

Der Arzt und Psychoanalytiker Alfred Lorenzer entwickelte in den späteren 1960er- und 1970er-Jahren seinen systematischen Entwurf zu einer materialistischen Sozialisationstheorie (Lorenzer 1972) und später zur Adaption des psychoanalytischen Erkenntnisprozesses in den Sozialwissenschaften im Rahmen der Tiefenhermeneutischen Kulturanalyse (Lorenzer 1986). Lorenzer ging es in der materialistischen Sozialisationstheorie primär um eine kritische Auseinandersetzung mit dem Biologismus der Psychoanalyse und den soziologisierenden Zugriffen auf die „Wahrheit der psychoanalytischen Erkenntnis“ (Lorenzer 1974) sowie um den Versuch, Freuds Triebe als etwas Leibliches – und damit als etwas zugleich Biologisches und Gesellschaftliches – auszuweisen.

In der Tiefenhermeneutischen Kulturanalyse begründete Lorenzer schließlich zum Ende seines Schaffens systematisch eine Methodologie der psychoanalytischen Sozialforschung. Lorenzer griff dabei auf seine frühen Überlegungen zur Rolle der Sprache im therapeutischen Behandlungsprozess und zum szenischen Verstehen zurück. Der Begriff des szenischen Verstehens zielte hier zunächst auf die Art und Weise des psychoanalytischen Verstehens als eines, das sich vom *logischen Verstehen* der bewussten Kommunikationsinhalte und auch dem *psychologischen Verstehen* der Emotionen eines Gegenübers unterscheidet. Denn, insofern Analytiker:innen im Behandlungsprozess unbewusst an der Lebenspraxis der Analysand:innen teilhaben, um in deren Erzählungen unbewusste Strukturen und Konfliktlagen zu erkennen, verstehen sie die Erzählungen als Konstellationen dramatischer Handlungen, als soziale *Szenen *einer Lebensgeschichte (Lorenzer 1970). Im Entwurf einer Tiefenhermeneutischen Kulturanalyse ging es nun darum, dieses szenische Verstehen ins Setting sozialwissenschaftlicher Forschung zu transponieren. Leitend war hier für Lorenzer stets die Frage nach der Wirkung kultureller Objektivationen (von Texten, Bildern, Filmen etc.) auf Rezipient:innen, die bewusst und unbewusst auf die im Material repräsentierte Inszenierung sozialer Interaktionen reagieren.

Zu den method(olog)ischen Prämissen der Tiefenhermeneutischen Kulturanalyse gehört generell zunächst die Grundannahme der Doppelbödigkeit jeglicher sozialen Interaktion zwischen menschlichen Subjekten. Im Anschluss an das oben skizzierte Verständnis der psychoanalytischen Methode als szenisches Verstehen fasst die Methodologie der Tiefenhermeneutik Texte und Bilder als „Gefüge von Szenen“ (König 2018, S. 29) auf, innerhalb derer bewusste und unbewusste „Lebensentwürfe – Ängste, Wünsche, Fantasien“ (König 2018, S. 29) – in einer spannungsreichen Beziehung zueinander inszeniert sind. Verstanden als präsentatives Symbolsystem (Lorenzer 1981, S. 30) wird sich diesem Szenengefüge über die affektiven Reaktionen von Interpret:innen auf den Text oder das Bild auf der Grundlage der Wirkung auf deren Erleben genähert. Die aus der Auseinandersetzung mit dem Datenmaterial entstehenden Assoziationen eröffnen einen Zugang zu den im Material inszenierten Lebensentwürfen und spannungsreichen Beziehungen. Methodisch leitend sind *Irritationen*, über die die Wahrnehmung der Interpret:innen gewissermaßen stolpert; Irritationen werden dabei nicht nur als Reaktion auf ein Aufscheinen von Ungereimtheiten verstanden, sondern als Verweis auf die dem Szenengefüge inhärente Doppelbödigkeit (König 2018, S. 31).

Tiefenhermeneutisches Interpretieren findet stets in Gruppen statt, denn nur in diesen können idiosynkratische Reaktionen auf das Material erkannt und korrigiert werden. Zudem gilt die praktische Prämisse, dass alle subjektiven Reaktionsweisen zwar als Gegenübertragungen auf das Material verstanden werden, dass dieser Zusammenhang jedoch immer auch nachvollziehbar an diesem Material plausibel gemacht werden und die Interpretation sich am Text beweisen muss. Allein der Hinweis auf eine Gegenübertragung wird im Kontext einer tiefenhermeneutischen Interpretation also nicht als hinreichend betrachtet.

In der Interpretation wird ausgehend von den Irritationen eine *szenische Rekonstruktion *des Materials entwickelt, die an den durch die Irritationen markierten Ungereimtheiten ansetzt und den Text von dieser aufscheinenden Doppelbödigkeit des analysierten Szenengefüges aus als sinnhaft zu rekonstruieren sucht. Zentral ist dabei auch, dass diese szenische Rekonstruktion zunächst *ohne* Rekurs auf klinische oder gesellschaftstheoretische Begrifflichkeiten, die der Interpretation verfrüht einen potenziell irreführenden Stempel aufdrücken würden, auskommt. Ist das Material anhand mehrerer interpretierter Szenen verdichtet, lassen sich die manifeste und die latente Sinnebene des Materials unterscheiden, aus deren spezifischer Spannung sich der Sinn des dem Material inhärenten Szenengefüges ergibt (Lorenzer 1986; König 2019; Lohl 2020).

Die letzten Schritte der Tiefenhermeneutischen Kulturanalyse betreffen die Vermittlung der szenischen Rekonstruktion mit gesellschaftstheoretischen Überlegungen. Erst im Anschluss daran erfolgt die Vermittlung mit gesellschaftstheoretischen Überlegungen (bei Lorenzer in erster Linie mit marxistischer Gesellschaftstheorie), die thematisch das Analysierte in einem gesellschaftlichen Kontext reflektieren und einordnen.

## Erkundungen der Coronaproteste

Um beispielhaft darzulegen, wie eine psychoanalytische Erkundung eines hoch aktuellen gesellschaftlichen Phänomens aussehen kann, wollen wir uns im Folgenden mit der Forschung zu den Protesten gegen die Coronamaßnahmen, in der bundesdeutschen Debatte auch bekannt als „Querdenken“, auseinandersetzen.

Unterschiedliche Fragen und Ebenen stehen dabei im Fokus: Untersucht wird *erstens*, welche zuweilen starken bewussten und unbewussten Gefühls- und Konfliktlagen die Pandemie und die Maßnahmen zu ihrer Einschränkung in den diesen Ausgesetzten produziert und reaktiviert. Die Coronaproteste, die Leugnung der gesundheitlichen Bedrohung durch die Pandemie, die Identifizierung von Schuldigen in verschwörungstheoretischer Form, die Vergemeinschaftung – das alles scheinen Möglichkeiten zu sein, Ängste abzuwehren, Wünsche und Aggressionen auszuleben und so den inneren Haushalt zu regulieren. Solche Mechanismen sind aus der Ressentiment- und Rechtsextremismusforschung wohl bekannt, allerdings weisen die Coronaproteste einige Züge auf, die sich von den bislang bekannten Agitationsmustern und rechten Bewegungen beträchtlich unterscheiden (Brunner et al. 2021a).

Wer sind nun, das wäre die *zweite Frage*, die Teilnehmer:innen der Coronaproteste? Soziologisch betrachtet haben wir es mit einer durchaus heterogenen Masse zu tun. Es lässt sich (obgleich zum Beispiel Frauen von der Krise aufgrund der erhöhten – geschlechtlich ungleich verteilten – Sorgearbeit sehr viel mehr betroffen sind; Villa 2020) keine klare Geschlechter- oder Klassenstruktur ausmachen, auch wenn es offenbar einen Überhang an Freiberufler:innen gibt und die (obere) Mittelschicht eher übervertreten ist. Auch politisch kommen die Teilnehmer:innen aus unterschiedlichen Ecken, aus linken und esoterisch-grünen sowie aus konservativen und rechtsextremen Milieus (Nachtwey et al. 2020; Brunner et al. 2021b). Aus psychoanalytischer Perspektive interessieren uns die lebensgeschichtlichen Hintergründe, die wir mittels biografischer Interviews erheben: Wie funktioniert bei den Protestteilnehmer:innen das Zusammenspiel zwischen den lebensgeschichtlich entstandenen Dispositionen, frühen Affekt- und Konfliktlagen, der unmittelbaren Erfahrung der Pandemie und der Verarbeitung mittels der Coronaproteste und der Ideologie der Querdenker:innen? Wir oszillieren hier bei der Analyse immer wieder zwischen der psychoanalytischen Rekonstruktion der individuellen Sinngebungen, Konfliktlagen und Verarbeitungswege und einem soziologischen Blick auf die spezifische gesellschaftliche Position und die materiellen und diskursiven Rahmenbedingungen, in die die innerpsychischen Prozesse eingebettet sind und die Erfahrungen wie Verarbeitungswege mitstrukturieren. Das individuelle soll als soziales Leid erfahrbar und begriffen werden (dazu zum Beispiel Knasmüller et al. im Druck; Domdey 2022).

*Drittens* wird der Diskurs der Coronaproteste genauer beleuchtet und nach den spezifischen manifesten und latenten Angeboten gefragt, welche politische Akteur:innen den Zuhörer:innen machen: die Redner:innen auf den Protesten, die Influencer:innen im Internet, die Organisationen in ihren Videos, auf Flugblättern oder Homepages, die den Coronaprotesten nahestehenden Parteien, Alternative für Deutschland (AfD) oder Freiheitliche Partei Österreichs (FPÖ), im Parlament. Diese Propagandaanalysen orientieren sich an der Studie von Löwenthal (1949) über Reden von faschistischen Agitatoren in den 1940er-Jahren in den USA. Er hatte gezeigt, wie diese stets ein gesellschaftlich produziertes Unbehagen aufgreifen und die Realität (die tatsächlichen gesellschaftlichen Problemlagen, die Situation der Zuhörer:innen, die Position und Macht der Agitatoren selbst) phantasmatisch über Prozesse der Spaltung, Verschiebung und Projektion so umarbeiten, dass der von den Agitatoren angeführte Kampf gegen die zu Schuldigen und Feinden Erklärten als ersehnter und einziger Ausweg erscheint. Während die Agitator:innen der Coronaproteste auf einer manifesten Ebene stets die Vernunft gegen den Irrsinn der „Panikmache“ und der „Coronadiktatur“ in Stellung bringen, werden einerseits über die Verleugnung der Pandemie und andererseits über bedrohliche Weltuntergangsszenarien Ängste und Konflikte verstärkt und zugleich verschoben, Aggressionsobjekte zur Verfügung gestellt und über Heldeninszenierungen und zuweilen Vernichtungsfantasien Reinigung- und Heilsversprechen gegeben (Henze 2022; Graage 2022). Im Zusammendenken dieser Propaganda-Analysen und der biografischen Interviews sehen wir dann wiederum, wie diese diskursiven Angebote und auch das Erlebnisangebot, das von den Protesten als realen Massenansammlungen und den Online-Kanälen der Coronaprotestbewegung als virtuellen Kollektiven ausgeht, von den einzelnen Individuen aufgegriffen, umgearbeitet und zur Bearbeitung der inneren Konfliktlagen genutzt wird.

### Fallbeispiel: Anna

Wir wollen im Folgenden am Beispiel eines biografisch-narrativen Interviews mit einer in etwa 40-jährigen Frau, der wir das Pseudonym Anna gegeben haben und die im Rahmen eines Forschungsprojekts zu ihrer Beteiligung an den Coronaprotesten in Österreich befragt wurde, den tiefenhermeneutischen Erkenntnisprozess und -gewinn nachzeichnen. Der Nachvollziehbarkeit halber steht am Beginn der verschriftlichten tiefenhermeneutischen Interpretationen die Rekonstruktion des *manifesten Gehalts *des zugrunde liegenden Materials. Zuallererst gehen wir demgemäß Selbstdeutung und Selbstentwürfen der Interviewpartnerin nach. Im Sinne Lorenzers bewegen wir uns hier auf der Ebene des logischen und psychologischen Verstehens.

Im fast zwei Stunden dauernden Interview erfahren wir viel darüber, welche Identitätsentwürfe Anna aus ihren biografischen Erfahrungen ableitet, die immer auch als nachträgliche Konstruktionen verstanden werden müssen. Sie schildert ein turbulentes Leben, in dessen Mittelpunkt ihre Mutter steht. Erst spät habe die Interviewte realisiert, dass diese an einer narzisstischen Persönlichkeitsstörung gelitten habe, wodurch sie erst habe einordnen können, „was [sie] als Kind da erlebt“ und „was das eigentlich bedeutet hat“. Rückwirkend kann Anna also – ganz im Sinne des Wortes – diagnostizieren, warum ihre Mutter ihre gesamte Familie manipuliert habe. Die Abnabelung von der Mutter gelingt nur unzureichend: Eher überstürzt, „jung und dumm“, wie sie urteilt, sei Anna nach abgeschlossener Berufsausbildung mit einem neuen Freund von Deutschland nach Österreich gezogen, wofür sie gar ihre Stelle aufgegeben habe. Die Beziehung sei jedoch schnell zerbrochen, und sie sei ohne Geld und mit einer Ausbildung, die in Österreich nicht anerkannt wird, in einer leeren Wohnung in der Nähe von Linz gestrandet. In dieser schwierigen Situation sei sie von ihrer Mutter „wieder eingeholt worden“: Als diese ihr die Küche ihres Bruders bringt, entscheidet die Mutter, mit ihrer Familie, Annas Schwester und dem nach einem schweren Arbeitsunfall arbeitsunfähigen Vater, ebenfalls nach Linz zu ziehen. Anna schafft es nicht, dem Zugriff ihrer Mutter zu entkommen: Sie sei in eine Wohnung mit Garten gezogen, die ihr zwar die Flucht aus der leeren Wohnung, die sie als „Betonbunker“ bezeichnet, ermöglicht, sie jedoch „dummerweise“, wie sie sagt, in die unmittelbare Nähe „dieses Drachens“ geführt habe. Zum einen wird die Mutter mit drastischen Zuschreibungen belegt und damit ihre Abscheulichkeit demonstriert, zum anderen begibt sich Anna von sich aus immer wieder in ihren Wirkungskreis, kann sich also nicht aus den manipulativen Fängen der Mutter lösen. Dem von ihrer Mutter kontrollierten Umfeld hält Anna ihr „kleines Hexenhaus“ entgegen, in dem sie es sich in ihrer „kleinen heilen Welt“ eingerichtet habe. Diese blieb noch prekär, als sie ihren heutigen Ehemann kennenlernt, denn sie blieb stets „greifbar in der Nähe“ der Mutter. Mit Walter sei sie eine glückliche Beziehung eingegangen und lebt heute zusammen mit ihm und ihren beiden gemeinsamen Kindern.

Zum endgültigen Bruch mit der Mutter kommt es erst, als diese einmal zu oft Kritik an den jungen Eltern übt, woraufhin Anna, wie sie sagt, zum ersten Mal in ihrem Leben für sich „aufgestanden“ sei, anstatt blind zu rebellieren oder wegzulaufen – seither sei sie für die Mutter „gestorben“. Zur Zeit des Interviews hat Anna bereits seit Jahren keinen Kontakt mehr zu ihrer Mutter. Auch mit ihrer Schwester und ihrem Vater, die sich aus ihrer Perspektive zu sehr von der Mutter manipulieren lassen, will Anna nichts mehr zu tun haben. Anna beschreibt die Abkopplung als kathartischen Prozess, der nach dem Tod ihrer geliebten Großmutter – ohne Therapie, wie sie stolz erzählt, aber mithilfe esoterischer Ideen und nach intensiver Selbstfindung – darin mündet, dass sie heute, viele Jahre später, ihren „inneren Frieden“ gefunden habe und große Dankbarkeit der Mutter gegenüber empfinde. Nach erfolgreicher äußerer Ablösung habe sie sich nun auch innerlich lösen und ihr mittlerweile verzeihen können. Diese Erfahrungen haben Anna erkennen lassen:

„dass das Wichtigste ist, die Verantwortung fürs Leben selbst zu tragen; also ich kann’s nicht wem andern überstülpen, meine schlechte Laune, oder was ich erlebt hab, meine Biografie, ich muss die Verantwortung für mich selbst übertragen, äh tragen.“

In Annas Erzählung geht die Abnabelung mit ihrer Selbstermächtigung einher: Die Zumutungen durch die Gängelung der Mutter haben sie gelehrt, die Verantwortung für ihr eigenes Leben zu tragen. Im gleichen Zug unterläuft Anna diesen Selbstentwurf jedoch durch eine Fehlleistung wieder, die – in Lorenzers Begrifflichkeit –* irritiert* und in der eine hier konflikthaft gewordene Doppelbödigkeit der erzählten Szene aufscheint: So ist durchaus zutreffend, dass sie in ihrer Sicht auf die Vergangenheit und die Welt bisweilen Verantwortung auch auf andere „überträgt“, wie im Folgenden noch zu zeigen sein wird. Die durch die Fehlleistung ausgelöste Irritation kann somit als erster Hinweis darauf gedeutet werden, dass sich, durch diese vermittelt, *latenter Bedeutungsgehalt* Ausdruck verschafft.

Die hier nur kursorisch referierte Einstiegserzählung und die darin eingelassenen manifesten Selbstentwürfe sind in mehrerlei Hinsicht aufschlussreich und dienen als Ausgangspunkt für die nun folgende *szenische *Rekonstruktion des Interviews. Aus den Schilderungen lernen wir über die manifeste Charakterisierung ihrer Mutter, die von Anna als manipulativ, verfolgend, narzisstisch gestört erlebt wird, mit der sie sich, angestoßen durch den Tod ihrer Großmutter, nach außen hin versöhnt zeigt. Wir erfahren auch von Annas Selbstdeutung als Person, die aus den Peinigungen gestärkt hervorgegangen sei und über ihr Leben nunmehr eigenmächtig bestimme. Als Tiefenhermeneut:innen nehmen wir die Entwürfe der Interviewpartner:innen zwar ernst, begegnen ihnen jedoch zugleich mit vorsichtiger Skepsis und dem erkenntnisleitenden Verdacht, dass die Erzählung doppelbödig ist und sich hinter deren Fassade Inhalte verbergen, die nichtsprachliche Form annehmen. Und tatsächlich regt sich angesichts der drastischen Sprache Zweifel an der Versöhnungserzählung, hinter der sich starke, scheinbar bedrohliche Affekte andeuten, die dethematisiert werden müssen. Eigene aggressive und verfolgende Anteile kehren in den Bildern der bedrohlichen Anderen wieder. Nicht zuletzt nähern wir uns mit der Methode dem szenischen Gehalt des Materials nicht nur über die Analyse dessen, *was* gesagt wird, und der sich darin auftuenden Widersprüche, sondern darüber, *wie *gesprochen wird: Das Aufnahmegerät lief bereits in etwa eine Stunde, als die Einstiegserzählung zum Erliegen kommt und der Interviewer mit immanenten Nachfragen erstmals zum Zug kommt. Die scheinbar nicht enden wollende Stehgreiferzählung hinterlässt bei diesem das Gefühl, überwältigt und eingeschnürt worden zu sein. Die subjektiven Eindrücke und Gefühlsreaktionen des Interviewers werden durch jene der Interpretationsgruppe gestützt; diese äußerte während der Interpretationssitzung Sorgen, dass der Interviewer überrollt worden sein könnte. In der Szene, die sich zwischen Interviewer und Interviewpartnerin entfaltet hat, reinszeniert sich Annas Verfolgungsgeschichte unter anderen Vorzeichen. Eingespannt in eine *latente* Dynamik ist es der Interviewer, der sich nunmehr von Anna eingeengt, verfolgt und *untergründig* bedroht fühlt.

Vorläufige Deutungen dieser Art verdichten sich zu einer stimmigen Interpretation des Falles, indem diese an weiteren irritierenden beziehungsweise zu jener ersten in einem irritierenden oder bestätigenden Verhältnis stehenden Szenen plausibilisiert werden. Aus Darstellungsgründen müssen wir uns hier auf eine ausgewählte Passage beschränken, an der Anna festmacht, warum sie ihren Rückzugsort – ihr „Hexenhaus“ – aufgeben musste:

„dann ist was anderes dann in mein Leben getreten, dass die Katze, die ich damals gehabt hab, die auch so’n Anker war, in der Zeit, wo ich alleine war, und hat mir mein Nachbar leider umgebracht, erschlagen, und im Garten rüber ins Wasser geworfen, den hab ich dann gefunden, und der Walter hat ihn dann aus dem Wasser rausgeholt, weil ich nicht rein konnte, wegen dem Bäuchlein.“

Wir können nur mutmaßen, was wirklich vorgefallen ist – die Erzählung, dass der Nachbar, ohne dass es eine konfliktreiche Vorgeschichte gab, plötzlich das arme Tier erschlug, wirkt wenig plausibel –, aber die Schilderung ist symptomatisch für Annas Wahrnehmungswelt: Auf der Suche nach einer heilen Welt ist sie umgeben von bösartigen Kräften, die ihr grundlos Schaden zufügen wollen. Die Doppelbödigkeit der Bezeichnung ihrer „kleinen heilen Welt“ als „Hexenhaus“ wird angesichts dieser Szene augenscheinlich: Zum einen will Anna damit das Heimelige des eigenen Heims betonen, deutet zum anderen jedoch unwillkürlich an, dass darin zugleich eine Hexe ihre Unwesen treibt und in einen Nachbarschaftskrieg verwickelt ist. Szenisch gelesen, verbergen sich darin auch Annas eigene aggressive, womöglich auch bedrohliche Anteile, die im gesamten Interviewverlauf nicht zur Sprache kommen: Die Hexen sind die Anderen.

Die Pandemie bricht nun in die Situation des „inneren Friedens“ ein, den Anna sich mit ihrer Familie geschaffen zu haben meint. Anna arbeitet zu diesem Zeitpunkt in Teilzeit im Gesundheitsbereich und studiert nebenbei, während ihr Ehemann, der ebenfalls in Teilzeit in der sozialen Arbeit beschäftigt ist, ihr tatkräftig bei der Sorgearbeit unter die Arme greift. Ihre Kindheitserfahrungen setzt Anna unmittelbar in Bezug zu ihrem politischen Aktivismus gegen die Regierungsmaßnahmen:

„das alles [seufzen] unterm Strich, is’ eigentlich das, was mich antreibt, dann weiterhin für die Gerechtigkeit, für alle, für alles aufzustehen; ja, also jetzt für meine Kinder, vor allen Dingen.“

In ihren Klagen gegen die staatlich dekretierten Einschränkungen sorgt sie sich neben ihren Klient:innen vor allem um ihre Kinder, denen die Isolierung schade und die sie besonders vor dem drohenden Impfzwang beschützen wolle. Im Interview spricht Anna sich vor ihrem eigenen Erfahrungshintergrund dafür aus, „dass jeder Mensch entscheiden und so selbstbestimmt und so frei wie möglich Verantwortung fürs eigene Leben mittragen darf“. Was für ihre Kinder am besten ist, weiß Anna hingegen offenbar ganz genau: Sie schreibt Briefe an die Direktion des Kindergartens ihrer Tochter, und zusammen mit ihrem Mann beschließt sie, ihre Tochter aus dem Kindergarten sowie ihren Sohn aus der Schule zu nehmen und zu Hause zu unterrichten. In der Schule sei unter den Kontaktbeschränkungen und der Maskenpflicht „die Möglichkeit zur freien Entfaltung“, die Anna ihren Kindern gerade deshalb bieten will, weil sie selbst von ihrer Mutter erdrückt wurde, nicht mehr gegeben.

Während die pandemiebedingten Einschränkungen in der Wahrnehmung von Anna allerorts für Spaltungen und Verwerfungen sorgten, seien die innerfamiliären Beziehungen in der Krise noch enger geworden. Zusammenhalt findet sie jedoch nicht nur bei ihrer Familie, sondern auch auf den Demonstrationen, von denen sie überschwänglich berichtet:

„des waren Familien; des waren Rollstuhlfahrer; es waren mit Rollator; es war und diese Energie, dieses miteinander Singen und für etwas Aufstehen; das war ja a positive Energie.“

Weder Hooligans noch sonstige rechte Gruppen habe sie wahrgenommen, die das Bild einer harmonischen, energetischen und familiär anmutenden Masse, aus der niemand ausgeschlossen wird, in Zweifel ziehen könnten.

Hinter den Beschwörungen familiärer Harmonie und nachträglicher Versöhnung mit der Mutter müssen wir hingegen dazu quer liegende Affektlagen vermuten, die mit den Selbstbildern unserer Interviewpartnerin im Konflikt stehen. Wir konnten bereits herausarbeiten, dass die überbordenden Aggressionen und Enttäuschungen, die Anna gegenüber ihrer Mutter zwar mit der neu entdeckten Dankbarkeit zudeckt, immer wieder aufbrodeln und auch im Interview selbst reinszeniert werden. Und auch, dass die Bürden der pandemiebedingten Einschränkungen friktionslos an der Kleinfamilie vorübergezogen sind, erscheint unplausibel – auch wenn ihre Wohnraumsituation mit Garten durch die Teilzeitbeschäftigung beider Eltern und die Unterstützung der im selben Haus lebenden Großmutter (Walters Mutter) – durchaus vorteilhaft gewesen zu sein scheint.

All die sich immer wieder durchschlagenden Spannungen und Aggressionen, die nicht ins eigene Selbst- und Familienbild passen, werden nun in Annas Erzählung an der Regierung verhandelt: Dieser und ihrer Corona-„Propaganda“ attestiert Anna manipulatives Verhalten, das ihr durch die eigene Mutter allzu vertraut sei und zu dessen Aufklärung sie sich insbesondere aufgrund dessen in der Lage sieht, und die Regierung ist es auch, die versucht, durch Abstandsregeln und Kontaktverbote einen Keil in Familien und die Gesellschaft als Ganze zu treiben. Diesen Manipulations- und Spaltungsversuchen hält Anna Aufrufe zu positiven, beschwichtigenden und einenden Botschaften entgegen: „liebe Regierung, propagier’ doch was anderes, geht’s spazieren, tut’s was Gutes, positive Nachrichten“. Aufrufe zu mehr Gelassenheit ziehen sich durch das ganze Interview und verstärken den Eindruck, dass diese auch dazu dienen, die eigenen aufkochenden Affekte in Schach zu halten. So soll die Pandemie zur Einladung umgedeutet werden, um wieder

„dankbarer [zu] sein, für das was wir haben, und mehr auf die andern schauen, die nicht so viel haben, und jeden so sein lassen, wie er is, also geh impfen, wenn du möchtest, tu das, aber bitte lass auch mich in Ruhe, wenn ich nicht möchte; ja und den Kindern genauso; bitte jeder, wie er möchte.“

Wie bereits der Tod ihrer geliebten Großmutter soll nun ein weiteres Unglück den Boden für mehr Dankbarkeit, Zusammenhalt und Solidarität bereiten: die Krise als Chance. Und diese Chance will Anna ergreifen, indem sie sich gegen die Drangsalierungen durch die Regierung zu Wehr setzt und für mehr Gerechtigkeit einsteht, wozu sie sich in Anbetracht ihrer einschneidenden Kindheitserfahrungen berufen und gewappnet fühlt. Als Beweggrund für ihr Aufbegehren führt Anna hingegen nicht ihre eigene Betroffenheit ins Feld – sie selbst hätte unter der Krise nicht zu leiden, sondern die unabsehbaren Folgen für die unmündigen Kinder und die kommenden Generationen motivierten ihren Protest. Auch hier ergibt sich eine szenische Nähe zu ihrer Erzählung über den Moment der endgültigen Ablösung von ihrer Mutter: Auch dieses „Aufstehen“ war eine Verteidigung ihres ersten Kindes, in dessen Erziehung die Mutter sich zu sehr einmischen wollte. Es bleibt dabei im Interview völlig unklar, was ihre Kinder tatsächlich von den Maskenbefreiungsattesten und von der Entscheidung, sie durch den Heimunterricht noch mehr zu isolieren, halten. Auch das Gemeinschaftserleben auf den Demonstrationen ist vor allem für Anna ein Erlebnisangebot – ihr Sohn begleitete die Eltern zwar auf einer der Kundgebungen, wurde jedoch dadurch eher verschreckt.

Im Kontrast zu den eingangs vorgestellten Passagen zeichnet sich nun der *latente* Gehalt des Interviews deutlicher ab; dieser steht zum manifesten Gehalt der Erzählung in einem signifikanten Spannungsverhältnis, das wiederum den Sinn der lebensgeschichtlichen Erzählung konstituiert: Anna sieht sich und ihre Familie von manipulativen, böswilligen Akteur:innen bedroht und sieht sich dadurch zur Rebellion gezwungen. Sie selbst und ihr soziales Gefüge spricht sie von Aggressionen, unliebsamen Affekten und Spannungen frei, adelt die Krisen sogar zur Triebkraft für die Stärkung des inneren Zusammenhalts und plädiert für Dankbarkeit und Mäßigung. Diese Selbstinszenierung lässt sich hingegen angesichts der immer wieder sich abzeichnenden Spannungen im Material nicht stützen. Es wird deutlich, dass sich darin Strategien verbergen, um brodelnde Affekte zu deckeln und zu dethematisieren, aber auch das eigene manipulative Vorgehen – gegenüber den Kindern und den Familienmitgliedern, die sie nicht mehr sieht, wenn sie nicht mit Annas Mutter brechen – und die daran verspürte Lust müssen kaschiert werden. Nicht zuletzt können wir den Fokus auf das angebliche Leid der Kinder vor diesem Hintergrund deuten: Hinter den berechtigten Sorgen um ihr Wohl steckt auch eine Abwehrstrategie, um einen Umgang mit den eigenen Ängsten – die gar nicht erst zur Sprache kommen dürfen – zu finden. Anna geht also selbst immer wieder manipulativ vor oder instrumentalisiert ihre Kinder für die eigenen Zwecke und Bedürfnisse, verortet solches Vorgehen hingegen ausschließlich bei den Politiker:inner und Medien.

### Theoretische Einordnung

In der szenischen Rekonstruktion manifester und latenter Sinninhalte und ihrem Spannungsverhältnis haben wir theoretische Überlegungen noch ganz bewusst ausgeklammert und so der Eigenlogik beziehungsweise Eigendynamik des szenischen Gehalts des Materials den Vorrang gegeben. Im nächsten Schritt geht es insbesondere darum, theoretisch zu begreifen, durch welche gesellschaftlichen Kräfte die latenten Affekt- und Konfliktlagen (mit-)hervorgebracht werden.

Der Aufsatzform halber können wir an dieser Stelle keine hinreichende theoretische Einordnung vornehmen, wollen jedoch Blickrichtungen skizzieren, die diesen letzten Schritt der tiefenhermeneutischen Interpretation erhellen. *Erstens *können wir in den unmittelbaren Krisenerscheinungen Katalysatoren vermuten, die Ängste wecken und projektiven Verarbeitungsmodi Vorschub leisten können. Die Verbreitung eines neuartigen Virus hat zunächst auch Anna geängstigt, aber insbesondere die politische Reaktion auf dessen rasche Verbreitung reaktivierte bei ihr biografisch verankerte Ängste, manipuliert, verfolgt und hintergangen zu werden. Die Affektqualität aktueller Konfliktkonstellationen – auch das ist für die psychoanalytische Sozialpsychologie charakteristisch – speist sich immer auch durch mitunter weit zurückliegende und verdrängte Erfahrungen, und diese prägen die Wahrnehmung der gegenwärtigen (Konflikt‑)Lagen.^1^*Zweitens *sind Biografie und gelebte Erfahrung der Interviewpartner:innen in gesellschaftlich strukturierte Entstehungsbedingungen eingelassen, weshalb es stets eines intersektionalen Blickes bedarf, der Klassenlage, Geschlecht und „race“ mitdenkt. Im vorliegenden Fall fällt insbesondere die vergeschlechtlichte Arbeitsteilung ins Auge, denn wir können annehmen, dass die Ausgangsbeschränkungen, das Homeschooling und die Zunahme an Care-Arbeit auch die in der Familie schlummernden Abhängigkeitskonflikte, die dethematisiert werden, verschärfen.^2^ Das Zurückgeworfenwerden auf das eigene Zuhause in der Pandemie befeuert wohl auch innerfamiliäre Spannungen, die der imaginierten Familienharmonie entgegenstehen. *Drittens *können wir nachzuzeichnen versuchen, wie Anna sich verschwörungstheoretischer Versatzstücke bedient, um einen Umgang mit den vielfältigen, bedrohlichen Gefühlen zu finden, die den stützenden familiären Zusammenhalt und ihr Selbstbild bedrohen. Die identifizierten Übeltäter:innen der Krise, allem voran die Regierung und die manipulativen Medien, beziehungsweise die vernachlässigten und bedrohten Kinder und Klient:innen in der Pflege dienen ihr als Projektionsflächen, mit deren Hilfe unbewusste Konfliktlagen *schiefgeheilt *werden können.

## Fazit

Ziel unserer Ausführungen war es zu zeigen, dass psychoanalytisch informierte Gesellschaftsanalysen äußerst fruchtbar sein können, wenn sie aus der therapeutischen Praxis gewonnene Begrifflichkeiten nicht einfach bruchlos auf die Gesellschaft übertragen, sondern der spezifischen Vermittlung von Psyche und Gesellschaft Rechnung tragen. Am vorgestellten Fallbeispiel wird deutlich, dass sich die Beteiligung an den Coronaprotesten und die Affinität für verschwörungstheoretisches Denken aus einem komplexen Zusammenspiel von biografisch erworbenen Konfliktlagen, aktuellen Krisenerscheinungen und der je individuell gelagerten sozialen und vergeschlechtlichten Position verstehen lässt. Diese an der tiefenhermeneutischen Kulturanalyse veranschaulichte Perspektive ermöglicht es einerseits zu verstehen, wie eine von Verschwörungstheorien geprägte Weltsicht es möglich macht, lebensgeschichtliche Konfliktlagen zu bearbeiten, und andererseits aus gesellschaftstheoretischer Sicht danach zu fragen, welche Kräfteverhältnisse diese Konflikte und die entsprechenden Verarbeitungsmodi überhaupt erst hervorbringen. Ein gesellschaftskritisch geschulter Blick sollte idealerweise auch verhindern, dass das Verstehen der Prozesse in ein Verständnis gegenüber ressentimentgeladenen politischen Haltungen kippt.

## Footnotes

1 Zum psychoanalytischen Konzept der Nachträglichkeit: Kirchhoff (2009).

2 Zur Rolle von Geschlecht auf den Coronaprotesten: Daniel et al. (im Druck).
